# A case report of ruptured ectopic pregnancy plus massive hemoperitoneum on a heterotrophic pregnancy in a resource-poor setting, Mbengwi, Cameroon

**DOI:** 10.11604/pamj.2021.39.52.18513

**Published:** 2021-05-19

**Authors:** Nzozone Henry Fomukong, Edwin Ngouagna, Mandeng Ma Linwa Edgar, Claude Ngwayu Nkfusai, Fala Bede, Frankline Sevidzem Wirsiy, Samuel Nambile Cumber

**Affiliations:** 1 Department of Medicine and Surgery, Faculty of Health Sciences University of Buea, Buea, Cameroon,; 2 Microhealth Global Medical Centre, Mbengwi, Cameroon,; 3 Department of Public Health, School of Nursing and Public Health, University of Kwa-Zulu Natal, Durban, South Africa,; 4 Cameroon Baptist Convention Health Services (CBCHS), Yaoundé, Cameroon,; 5 Department of Public Health and Hygiene, Faculty of Health Sciences, University of Buea, Buea, Cameroon,; 6 Cameroon Society of Epidemiology (CaSE), Yaoundé, Cameroon,; 7 Faculty of Health Sciences, University of the Free State, Bloemfontein, South Africa,; 8 Institute of Medicine, Department of Public Health and Community Medicine (EPSO), University of Gothenburg, Box 414, SE - 405 30 Gothenburg, Sweden

**Keywords:** Heterotopic, rupture, ectopic, pregnancy, case report

## Abstract

Heterotopic pregnancy is a rare obstetrics phenomenon and carries significant maternal morbidity and mortality due to the risk of rupture of the ectopic pregnancy. Physicians tend to feel comfortable and relieved when an intrauterine gestation sac is seen. This results in an inadequate inspection of the adnexae and remaining structures during emergency bedside ultrasound despite a strong initial clinical suspicion of ectopic pregnancy. We present a case report of ruptured ectopic pregnancy and massive hemoperitoneum in a patient with heterotopic pregnancy. The diagnosis was done on bedside ultrasonography in a clinically unstable 32-year-old patient with a history of infertility. She presented with acute abdominal pain, body weakness, and amenorrhea. She underwent emergency laparotomy and salpingectomy. In our context where ultrasound is not readily available, practitioners carrying out salpingectomy for ruptured ectopic pregnancies should bear in mind the plausibleness of heterotopic pregnancy to properly handle the uterus.

## Introduction

Heterotopic pregnancy is the coexistence of intrauterine pregnancy (IUP) and extrauterine gestation [1, 2]. It is a rare occurrence; it was first reported in 1708 by Duverney as an incidental finding of intrauterine pregnancy while doing an autopsy of a patient who died due to ruptured ectopic pregnancy (Duvernay cited by Diallo *et al*.) [3]. It is a rare and dangerous life-threatening situation that is difficult to diagnose and easily missed [2]. The incidence in the general population is estimated to be 1 in 30,000, while a rate as high as 1 in 8,000 has been reported [4, 5]. The fallopian tube is the site of most of the ectopic implantation in heterotopic pregnancies, but the cervix or abdomen can also be involved [4, 6, 7]. However, the spontaneous occurrence is extremely rare, and the diagnosis and management require a high index of suspicion and delicate handling for an improved obstetrical outcome of on-going viable intrauterine pregnancy. This is a case report eliciting a similar occurrence of spontaneous heterotopic pregnancy and the subsequent obstetrical outcome of the patient following management in a resource-poor setting.

## Patient and observation

### Case presentation

A 31-year-old G_2_2P_2002_ with a relevant history of secondary infertility for 12 years presented with unknown duration of amenorrhea, severe lower abdominal pain, vomiting, and generalised body weakness of 3-day duration. She was unable to present at the hospital earlier because of the ongoing civil war and shutdown of town. On review of systems, she had headache, dizziness, per vaginal bleeding. On physical examinations, she was in an altered general state, asthenic, disoriented, pale, extremities cold clammy, pulse thready. Blood pressure 74/49 mmHg, pulse 132 bpm, SPO_2_90%, temperature 38°C. Abdomen looked swollen with generalised tenderness, dull on percussion, reduced bowel sounds. Vaginal exams, cervix was closed, cervical motion tenderness, and left adnexal tenderness.

### Diagnostic assessment

**Figure 1:**
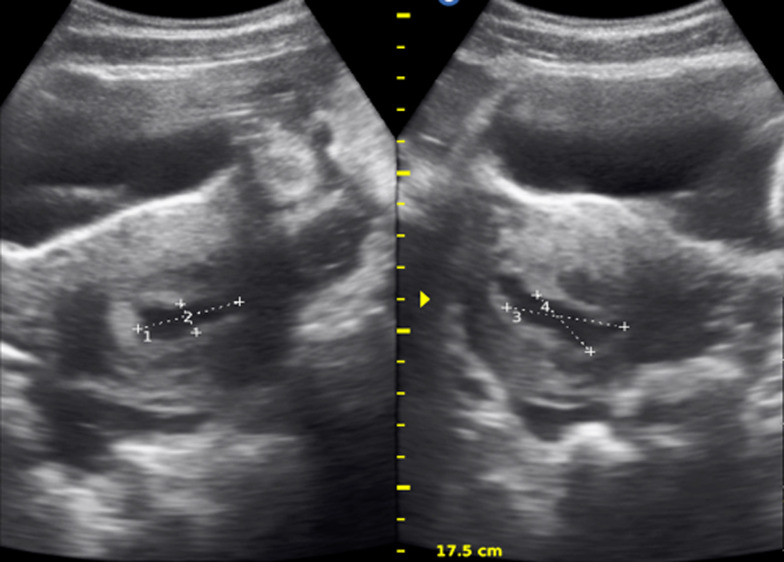
ultrasound image showing an intrauterine gestational sac at 7 weeks 2 days, free fluid at the pouch of Douglas

**Figure 2:**
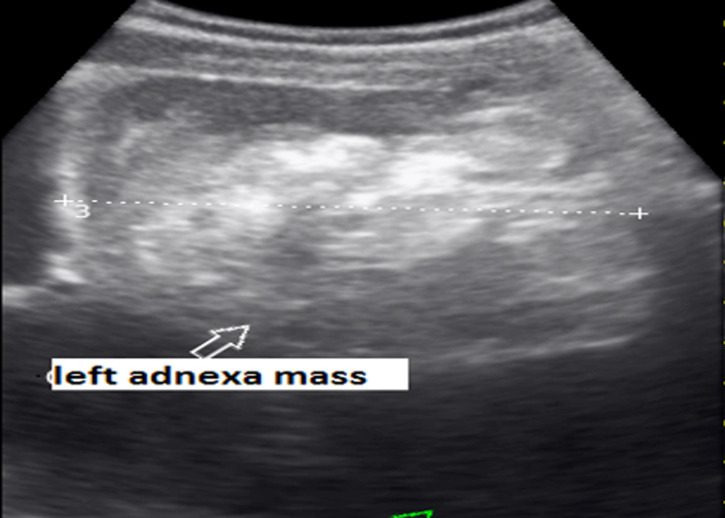
ultrasound image showing a complex left adnexal mass 12 cm x 10 cm

Urine pregnancy test done was Positive, Hb 6.9 g/dl. Urgent bedside ultrasound showed intrauterine pregnancy at 7 weeks 2 days´ gestational sac, presence of cardiac activity, plus mark-free pelvic exudate predominantly in pouch of Douglas (Figure 1), left complex adnexal mass 12 x 10 cm (Figure 2). After the above findings on ultrasound, a Paracentesis was done and collected 10cc of bright red blood. We concluded on hemoperitoneum plus probable ruptured left ovarian cyst. Laboratory was alerted to cross match and prepare two pines of O rhesus positive blood.

### Therapeutic intervention

**Figure 3:**
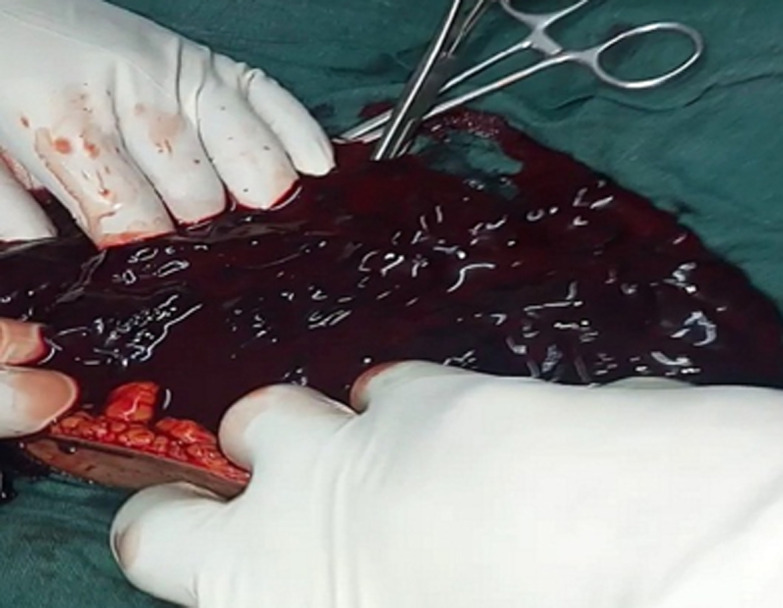
intraoperative finding of massive hemoperitoneum

The patient was rushed to the theatre for emergency laparotomy under general anaesthesia. A Pfannenstiel incision was done. Intraoperative findings were massive hemoperitoneum (Figure 3) with many clots estimated at 3.5 litres, ruptured left fallopian tube, left haemorrhagic ovarian cyst. Procedures performed were suction and removal of clots, left salpingectomy, and left cystectomy. The patient received a total of two pines of blood placed on 2500cc of fluids (alternating ringers lactate, 0.9% normal saline, and glucose 5%). On day 3 post laparotomy patient complained of severe lower abdominal pain and per vaginal bleeding. Her control Hb was 8.6 g/dl. She underwent suction dilatation and curettage (D&C) for incomplete abortion. A moderate amount of product of conception (POC) was collected during D&C. She was discharged on day 6 post-operation with no complains.

## Discussion

Heterotopic gestation, although fairly common with assisted reproductive techniques, is very rare in natural conception [8]. There are two categories of risk factors for heterotopic pregnancy: risk factors of ectopic pregnancy (history of infertility, sexually transmitted infection, intrauterine device, smoking, hormonal contraception, pelvic surgery) and ovulation induction and assisted reproductive technologies (ART) [4]. Our patient had a long history of infertility (12 years) as the only risk factor. The patient´s symptoms are often very similar to an ectopic pregnancy and they typically present with abdominal pain that may be localized or diffuse associated with vaginal bleeding or spotting. An adnexal mass or an enlarged uterus might be felt on pelvic examination. Depending on the stage of illness the patient may hypotensive or hemodynamically unstable. There are no physical exam/lab findings that are specific for heterotopic pregnancy but this diagnosis should be considered in any hypotensive pregnant patient with abdominal pain and an IUP identified on bedside ultrasound, especially in the setting of free fluid on ultrasound and/or history of ART [9]. Nearly 50% of the cases present with rupture, hemorrhage, and emergency intervention. Despite this, 2 in 3 patients presenting with a viable IUP have a chance of producing a living child if the diagnosis is made and treated appropriately [10]. Careful manipulation of the uterus is essential during laparotomy for the effective survival of intrauterine pregnancy.

## Conclusion

Heterotopic gestation, although common with assisted reproductive techniques, is very rare in natural conception [8]. There are two categories of risk factors for heterotopic pregnancy: risk factors of ectopic pregnancy (history of infertility, sexually transmitted infection, intrauterine device, smoking, hormonal contraception, pelvic surgery) and ovulation induction and assisted reproductive technologies (ART) [4]. Our patient had a long history of infertility (12 years) as the only risk factor. The patient´s symptoms are often very similar to an ectopic pregnancy and they typically present with abdominal pain that may be localized or diffuse associated with vaginal bleeding or spotting. An adnexal mass or an enlarged uterus might be felt on pelvic examination. Depending on the stage of illness the patient may hypotensive or hemodynamically unstable. There are no physical exam/lab findings that are specific for heterotopic pregnancy but this diagnosis should be considered in any hypotensive pregnant patient with abdominal pain and an IUP identified on bedside ultrasound, especially in the setting of free fluid on ultrasound and/or history of ART [9]. Nearly 50% of the cases present with rupture, hemorrhage, and emergency intervention. Despite this, 2 in 3 patients presenting with a viable IUP have a chance of producing a living child if the diagnosis is made and treated appropriately [10]. Careful manipulation of the uterus is essential during laparotomy for the effective survival of intrauterine pregnancy.
